# Switching Between Reference Adalimumab and Biosimilars in Adult Patients With Inflammatory Bowel Disease: A Systematic Literature Review

**DOI:** 10.7759/cureus.110943

**Published:** 2026-06-16

**Authors:** Shuroog Nabhan, Hesham Qary, Abdullah Ajwah, Bassam Alsaeedi, Yazeed Ajwa, Sara Alhashmi, Alia Hijazi, Jomana Akbar, Maha Asiri

**Affiliations:** 1 Gastroenterology, King Fahd Armed Forces Hospital, Jeddah, SAU; 2 Internal Medicine, King Fahd Armed Forces Hospital, Jeddah, SAU; 3 Faculty of Medicine, King Abdulaziz University, Jeddah, SAU

**Keywords:** bioequivalence, biologic therapy, clinical outcomes, cost-effectiveness, disease control, drug monitoring, immunogenicity, interchangeability, non-medical switching, treatment persistence

## Abstract

Biosimilar adalimumab agents have been introduced as cost-effective alternatives to reference adalimumab for inflammatory bowel disease (IBD); however, uncertainties remain regarding the outcomes of switching between reference and biosimilars in routine practice. Therefore, this systematic review was conducted to assess outcomes following switching between reference adalimumab and biosimilars in adult patients with IBD. A systematic search was undertaken in PubMed/MEDLINE, the Cochrane Library, and Google Scholar to identify relevant studies. Eligible studies included randomized controlled trials, cohort studies, registry-based analyses, and real-world evidence evaluating adult IBD patients (≥18 years) who switched between reference and biosimilar adalimumab. A total of seven studies comprising 5721 patients were included. Among these, three were prospective studies, three were retrospective studies, and one was a cross-sectional study. Follow-up ranged from six months to 26 months. Across studies, remission and response rates after switching were comparable to continuation on reference adalimumab, with most cohorts reporting remission in 74-90% of patients. Biomarkers generally remained stable or improved. Persistence outcomes were heterogeneous, with specialized centers reporting high multi-year persistence (>80%). Safety profiles were similar across products, with injection-site reactions being the most frequent adverse events (AEs) and a common cause of discontinuation. Serious AEs were rare. Immunogenicity data were limited but did not indicate clinically meaningful differences between reference and biosimilars.

## Introduction and background

Inflammatory bowel disease (IBD), including Crohn’s disease and ulcerative colitis (UC), is a chronic, relapsing inflammatory disorder of the gastrointestinal tract. IBD incidence and prevalence have risen sharply over recent decades, with millions of people currently living with IBD globally [1]. In Western Europe, for example, roughly 0.4% of the population, equivalent to 2.5-3 million individuals, is estimated to have IBD [2]. The diseases often begin in young adulthood and follow a course of repeated flares and remissions. Clinically, IBD causes symptoms such as abdominal pain, diarrhea, bleeding, and fatigue, and it can involve extra-intestinal complications such as arthritis and skin inflammation [3]. These factors contribute to substantial morbidity. Economically, IBD imposes a heavy burden as its chronic nature requires long-term medical care, often with expensive therapies. In Europe, the cost of healthcare systems in managing IBD amounts to 4.6-5.6 billion euros per year [2]. Apart from these indirect costs due to lost work, reduced quality of life further amplifies the impact. Current management of IBD is guided by disease severity and location. Mild UC can often be controlled with 5-aminosalicylic acid agents, such as sulfasalazine or mesalamine, and rectal therapies [4]. Moderate-to-severe IBD usually requires corticosteroids for induction of remission, followed by maintenance therapy with immunomodulators such as azathioprine, 6-mercaptopurine, methotrexate, or targeted biologic agents [5]. The advent of biologics has transformed care for patients with refractory or extensive disease. In particular, tumor necrosis factor-alpha (TNF-α) inhibitors achieve substantially higher rates of clinical remission and mucosal healing than traditional therapies [6]. Two tumor necrosis factor (TNF) inhibitors are widely used in IBD, including infliximab, an intravenous chimeric monoclonal antibody, and adalimumab, a fully human monoclonal antibody [7]. These agents have markedly improved outcomes, reducing the need for surgery and hospitalizations in many patients [7]. However, there are certain limitations of these agents as well. Biologics generally must be given continuously to maintain remission [8], and their long-term use carries safety considerations.

Adalimumab (Humira) is a fully human IgG1 monoclonal antibody that binds and neutralizes TNF-α. It was approved in the early 2000s as a treatment for moderate-to-severe Crohn’s disease and later for UC, particularly for patients who failed or could not tolerate infliximab [9]. Adalimumab dampens gut inflammation by targeting both soluble and transmembrane TNF-α. In pivotal clinical trials such as the CLASSIC (Clinical Assessment of Adalimumab Safety and Efficacy Studied as Induction Therapy in Crohn’s Disease) [10] and CHARM (Crohn's Trial of the Fully Human Antibody Adalimumab for Remission Maintenance) [11] for Crohn’s disease and the ULTRA (Ulcerative Colitis Long-Term Remission and Maintenance With Adalimumab) trial for UC [12], adalimumab significantly improved clinical remission and maintenance of response compared with placebo. Despite its advantages, adalimumab therapy has certain drawbacks. Cost is the most prominent issue. As a biologic protein drug, adalimumab is extremely expensive, with costs reaching thousands of dollars per person per year [13]. Such high prices create barriers to patient access and impose enormous strain on healthcare budgets. Safety is another concern. Adalimumab, like all TNF inhibitors, is associated with risks of serious infections such as reactivation of tuberculosis or hepatitis B and other immune-mediated adverse effects [14].

Adalimumab biosimilars have been developed to address precisely these needs. A biosimilar is a biologic product that is highly similar to an approved reference biologic in structure, function, and clinical effect [15]. The first adalimumab biosimilar was approved in Europe in March 2017 [16]. Since then, multiple companies have launched adalimumab biosimilars worldwide. In the United States, the first adalimumab biosimilar (Amjevita) was approved in 2016, and in 2021, the Food and Drug Administration (FDA) began designating certain adalimumab biosimilars as interchangeable [17]. Efficacy and safety outcomes have likewise been reassuring. In the largest prospective IBD study to date, the ADA-SWITCH (Adalimumab SWITCH) cohort followed several hundred adult IBD patients in remission [18]. Roughly half were transitioned to an adalimumab biosimilar and half continued on originator Humira, and the patients were monitored for one year. The results showed virtually identical outcomes between the groups [18]. Given these developments, a systematic review of the literature on adalimumab biosimilar switching in IBD is timely and important. Numerous recent studies have directly examined outcomes after switching in adult IBD patients [18,19]. Therefore, this systematic review aimed to assess the safety, efficacy, and immunogenicity of switching between reference adalimumab and its biosimilars in adult patients with IBD.

## Review

Methods

This systematic review was conducted according to a predefined protocol and adhered to the Preferred Reporting Items for Systematic reviews and Meta-Analyses (PRISMA) 2020 reporting guidelines [20].

Search Strategy

A comprehensive literature search was conducted in PubMed/MEDLINE, the Cochrane Library, and Google Scholar. The search covered studies published from January 2017 up to September 2025. Boolean operators and medical subject headings (MeSH) terms were applied to combine keywords: (“adalimumab” OR “reference adalimumab” OR “biosimilar adalimumab”) AND (“inflammatory bowel disease” OR “Crohn’s disease” OR “ulcerative colitis” OR “IBD”) AND (“switch” OR “switching” OR “non-medical switch” OR “interchangeability”) AND (“efficacy” OR “safety” OR “immunogenicity” OR “treatment outcomes” OR “real-world data”) AND (“observational study” OR “randomized controlled trial” OR “cohort study” OR “registry”). Reference lists of included articles and relevant reviews were hand-searched for additional eligible studies. 

Study Eligibility

Eligibility criteria were defined according to the Population, Intervention, Comparator, and Outcome (PICO) framework [21]. The population included adults (≥18 years) with a confirmed diagnosis of an IBD (Crohn’s disease or UC). The intervention of interest was switching from reference adalimumab to a biosimilar or vice versa. The comparator was continuation on either reference or biosimilar adalimumab without switching. Eligible outcomes included clinical remission or response rates, safety events, immunogenicity, treatment persistence, and cost-effectiveness data. Randomized controlled trials, prospective and retrospective cohort studies, case-control studies, cross-sectional studies, and registry-based real-world evidence studies published from 2017 onward, corresponding to the first approval of adalimumab biosimilar in Europe, were eligible for inclusion. Furthermore, full-text publications in English were eligible. Case reports, editorials, narrative reviews, letters, conference abstracts without full text, animal studies, and studies involving non-IBD patients or those exposed to other biologics in addition to adalimumab were excluded. 

Study Selection and Data Extraction

Two reviewers independently screened titles and abstracts against the eligibility criteria. Full texts of potentially relevant studies were retrieved and assessed in duplicate. Reasons for exclusion at the full-text stage were documented. Discrepancies were resolved by discussion or arbitration by a third reviewer. After that, data were extracted independently by two reviewers using a standardized form. Extracted variables included study characteristics, patient demographics, details of switching, clinical outcomes, safety and immunogenicity, treatment persistence, and quality of life outcomes where reported.

Outcomes

The primary outcomes were clinical remission and response after switching versus continuing adalimumab treatment. Secondary outcomes included objective biomarkers such as C-reactive protein (CRP) and fecal calprotectin (FC), mucosal healing, adverse events (AEs), serious AEs, immunogenicity measures, treatment persistence, hospitalization, and surgery outcomes.

Risk of Bias Assessment

Risk of bias was assessed independently by two reviewers. Observational studies were assessed using the Newcastle-Ottawa Scale (NOS) [22]. The NOS checklist has eight questions spread across three categories, which examine how well the study cohort represents the general population, whether confounding factors are controlled, and any biases in measuring outcomes. The single cross-sectional study was assessed using the Joanna Briggs Institute (JBI) checklist for cross-sectional studies to appraise methods for sampling, measurement of exposure and outcomes, and handling of confounding [23]. Disagreements were resolved by consensus or by consultation with a third reviewer.

Data Synthesis

Given the substantial clinical and methodological heterogeneity among included studies - encompassing variation in study design (prospective cohort, retrospective cohort, cross-sectional), patient populations (Crohn’s disease vs. UC, bio-naïve vs. biosimilar-experienced), biosimilar products assessed (SB5, ABP501, GP2017, MSB11022), duration of follow-up (6-26 months), and definitions of clinical remission - formal meta-analytic pooling was deemed inappropriate. A narrative synthesis was therefore used to integrate and contextualise findings across studies, in accordance with the Synthesis Without Meta-Analysis (SWiM) reporting guidelines [24].

Results

Included Studies

**Figure 1:**
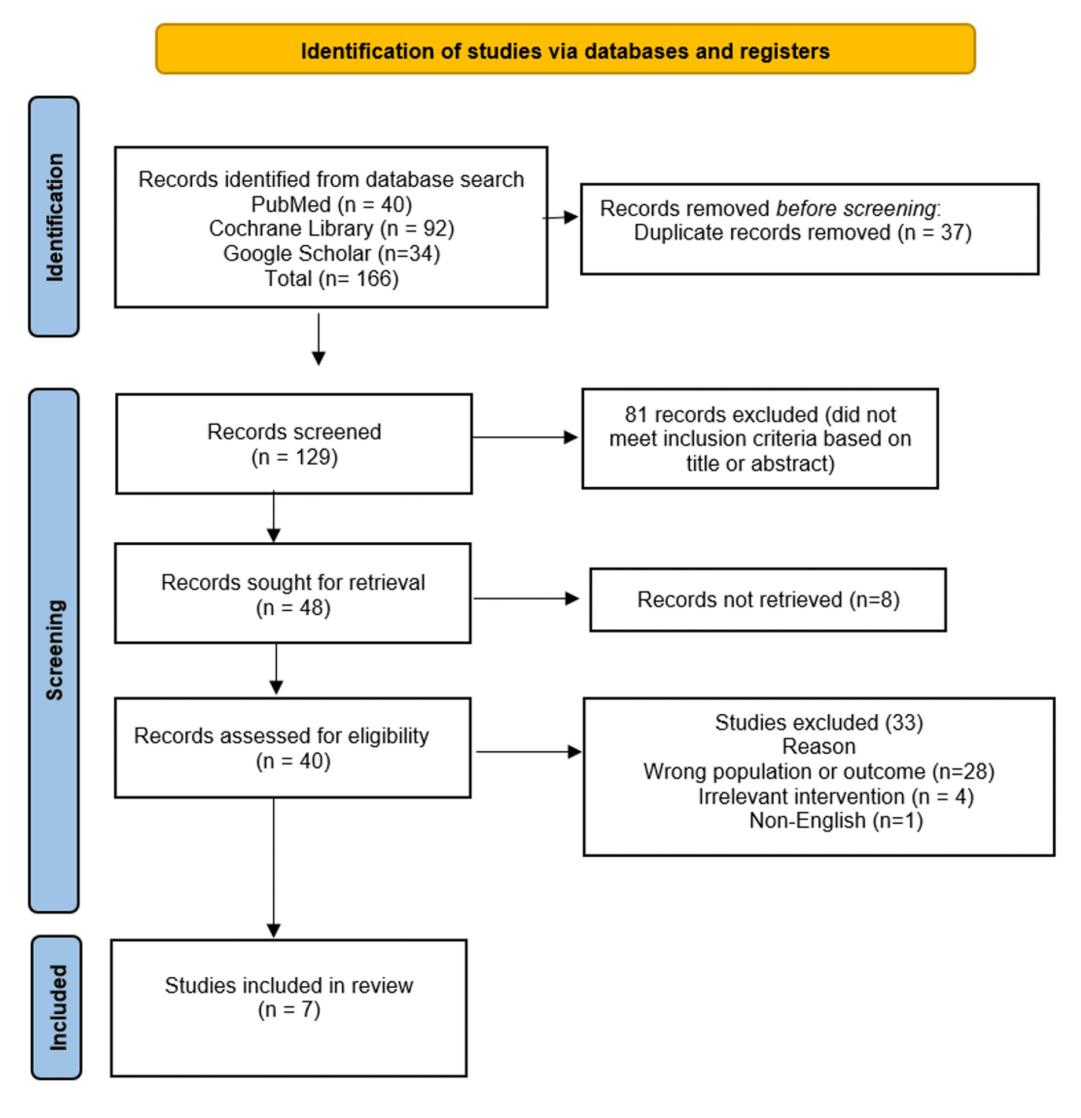
PRISMA flow diagram of this systematic review PRISMA: Preferred Reporting Items for Systematic Reviews and Meta-Analyses

The literature search provided 166 studies from PubMed (n=40), the Cochrane Library (n=92), and Google Scholar (n=34). Prior to screening, 37 duplicate records were removed. During the title and abstract screening, 81 records were excluded. A total of 40 articles were then sought and assessed for eligibility during the full-length screening. Following a thorough assessment of these full-length articles, seven articles were included in this systematic review [19,25-30]. The PRISMA flow diagram is illustrated in Figure 1.

Study Characteristics

A total of 5721 patients were included in the seven studies. Among these, three were prospective studies [19,25,28], three were retrospective studies [27,29,30], and one was a cross-sectional physician survey study by Jin et al. [26]. Follow-up ranged from six months to 26 months. 

The dominant clinical trend in the literature indicates that adalimumab biosimilars achieve remission and response rates broadly comparable to those of the reference product in both biologic-naïve patients and non-medical switchers. High absolute rates of maintained clinical remission after transitioning to or initiating a biosimilar were consistently reported, with figures remaining in the mid-70s to high-80s percent range for most patient cohorts [19,25,27,29]. Specifically, clinical efficacy and persistence outcomes were thoroughly evaluated by Tapete et al. [19] and Cingolani et al. [25]. Point-in-time clinical remission and high physician satisfaction were demonstrated in the cross-sectional survey by Jin et al. [26]. Broad therapeutic success across multiple biosimilar brands was also highlighted in the large multicenter cohort by Tursi et al. [27]. In contrast to these positive clinical outcomes, long-term treatment persistence and drug survival displayed notable heterogeneity across different clinical settings. While Lukas et al. [28] observed a significantly lower long-term drug persistence with the SB5 biosimilar compared to the originator product, a retrospective retail pharmacy claims database analysis by Jin et al. [29] demonstrated moderate overall 12-month persistence rates in unselected real-world populations. Conversely, excellent long-term drug survival was found by Liu Chen Kiow et al. [30], who reported no statistically significant differences in 30-month multi-year persistence between patients mandatorily switched to biosimilars and those remaining on the reference product. Objective inflammatory biomarker trends, such as CRP and FC, were reported less uniformly but generally pointed toward stability or modest objective improvement post-switch. Cingolani et al. [25] reported little biomarker change overall but a decrease in CRP-positivity for some patients on SB5, while Lukas et al. [28] observed a transient elevation in FC at week 26 in the SB5 arm that equalized by week 52. However, Tapete et al. did not find any significant difference regarding CRP between groups [19]. Persistence and drug survival showed heterogeneity. For example, Jin et al. reported only moderate 12-month persistence [29]. Liu Chen Kiow et al. reported 30-month persistence of 83.7% for originator compared to 88.2% among biosimilars (p=0.451) [30]. However, Lukas et al. found significantly lower long-term persistence with SB5 compared with originator adalimumab [28], and Tapete et al. [19] also reported high persistence in switchers. Safety reporting consistently highlighted local injection-site reactions (e.g., pain, pruritus, or erythema) as the most frequent and clinically relevant tolerability issues, serving as a recurrent driver of drug discontinuation across multiple cohorts [19,27,28]. Structured immunogenicity testing was notably sparse across the included real-world evidence [19]. Objective endpoints beyond clinical symptoms - such as systematic mucosal healing, steroid-free remission, hospitalizations, and surgical rates - were evaluated less uniformly [27]. 

The comprehensively extracted data, clinical endpoints, and detailed design parameters for each included study are systematically organized in Table 1.

**Table 1:** Study characteristics and outcomes AE: adverse event; CRP: C-reactive protein; FC: fecal calprotectin; ISP: injection-site pain; ADA: adalimumab; UC: ulcerative colitis; CD: Crohn’s disease; IBD: inflammatory bowel disease; mo: months; wk: weeks; KM: Kaplan-Meier; UBC: University of British Columbia; LOR: loss of response; UK: United Kingdom; RP: reference product; HBI: Harvey-Bradshaw Index; PS: propensity score; TNFi: tumor necrosis factor inhibitor; IQR: interquartile range

Study	Design	Setting/population	N (groups)	Intervention/ comparator	Follow-up	Key outcomes/results (brief)	Notable safety/immunogenicity	Limitations/notes
Tapete et al. (2022) [19]	Prospective study	Patients with histologically confirmed IBD (UC or CD)	146 total; 48 ADA-naïve; 98 switched from originator to SB5.	SB5 biosimilar: (1) first-line adalimumab in naïve; (2) non-medical switch originator → SB5.	12 mo	Naïve (n=48): remission 3 mo 81.3%, 6 mo 72.9%, 12 mo 90.6%; dose escalation 8.3%. FC decreased significantly at baseline → 3/6/12 (p<0.001). CRP decreased at 6 and 12 mo. Switchers (n=98): remission 3 mo 97.9%, 6 mo 86.7%, 12 mo 74.5%; dose escalation 9.18%. Persistence higher in switchers. FC and CRP were not significantly different at endpoints.	AEs 36.3%; ISP 24.7% - mostly in switchers (88.9%) (p=0.001). 3 severe AEs led to discontinuation. No significant difference between groups after adjusting for ISP.	Observational, regional registry; small immunogenicity sample.
Cingolani et al. (2021) [25]	Multicenter prospective study	IBD patients on ADA originator at 4 Italian centers (Padua Coordinating Center; Santorso, Pisa, Genoa)	55 switched to ABP501; 25 switched to SB5; 38 matched non-switch controls (reported groups).	Non-medical switch to ABP501 or SB5 vs continuing ADA originator.	6 mo	ABP501 (n=55): remission 85.5% → 76.4% at 6 mo (p=0.09); median FC 53 µg/g → 50 µg/g (p=0.90); steroid use increased (p=0.01); 8/55 (14.5%) discontinued (6 ineffectiveness, 1 AE, 1 surgery). KM drug survival vs non-switch p=0.20. SB5 (n=25): remission 96% → 84% (p=0.20); FC 97 µg/g → 50 µg/g (p=0.20); CRP positives decreased (p=0.03); 1 AE discontinuation.	ABP501: 1 discontinuation for AE; SB5: 1 AE discontinuation.	Short follow-up (6 mo); small SB5 sample (n=25).
Liu Chen Kiow et al. (2024) [30]	Single-centre retrospective observational study	Tertiary IBD centre (St. Paul’s Hospital/UBC).	271 total; 228 biosimilar switchers; 43 originator controls.	Mandatory switch from Humira → multiple biosimilars (Idacio, SB5/Hadlima, Hulio, Hyrimoz, Amgevita) vs continuing Humira.	Up to 30 mo (primary outcome: 30 mo persistence); mean post-switch durations 24 mo	Persistence at 30 mo: originator 36/43 (83.7%) vs biosimilars 201/228 (88.2%) (p=0.451). KM log-rank p=0.543 (no significant difference). Discontinuations: originator 7 (16.3%) vs biosimilars 27 (11.8%). Per-biosimilar discontinuations: Idacio 2/27 (7.4%), SB5 4/63 (6.35%), Hulio 19/86 (22.1%), Hyrimoz 2/48 (4.17%), Amgevita 0/4.	LOR discontinuations: originator 2 (4.65%) vs biosimilars 14 (6.11%). AE discontinuations: originator 2 (4.65%) vs biosimilars 10 (4.39%). Hospitalizations for flare: originator 5 (11.6%) vs biosimilars 13 (5.7%).	Retrospective, single-centre; mandatory policy context; heterogeneous biosimilars pooled.
Jin et al. (2024) [26]	Cross-sectional, point-in-time physician survey	Gastroenterologists and consenting patients in France, Germany, Italy, Spain, UK	375 patients receiving ABP501 at consultation: 239 ABP501 initiators; 136 RP → ABP501 switchers. Reference cohort: 329 RP initiators.	ABP501 as first-line advanced therapy (initiators) or direct switch from RP → ABP501 (switchers); RP initiators as reference.	Point-in-time (median treatment duration reported: initiators 7.5 mo; RP → ABP501 switchers 7.7 mo)	ABP501 initiators (n=239): physician-assessed clinical remission at consultation 177/239 (74.1%); CD HBI remission 84.7%, UC Mayo remission 57.4%. RP → ABP501 switchers (n=136): physician-assessed remission 121/136 (89.0%); CD remission 87.5%, UC remission 80.6%.	No detailed safety/adverse event reported. High physician satisfaction: 91.6% (initiators) and 98.5% (switchers).	Cross-sectional, point-in-time (no longitudinal outcomes).
Lukas et al. (2022) [28]	Prospective multicentre study	Adult patients with CD on maintenance originator adalimumab	207 eligible (175 CD). Switched: 74 → SB5; continued originator: 101. After exclusions: 129 CD available (56 SB5, 73 originator). PS-matched analysis: 54 SB5 vs 54 originator.	Switch from originator-adalimumab → SB5 vs continuing originator adalimumab.	Up to 104 wk (data available at wk 10, 26, 52, 78, 104)	HBI: matched baseline HBI 3.2 in both cohorts; mean HBI remained <5 at all reported timepoints; differences not clinically meaningful through wk 52. CRP: no clinically meaningful differences through wk 52. FC: baseline similar (203 vs 214); at wk 26, SB5 cohort had higher mean FC (410 vs 140; significant difference), but by wk 52 no meaningful difference. Treatment persistence: wk 26 originator 0.96 vs SB5 0.89; wk 52 originator 0.87 vs SB5 0.65; wk 104 originator 0.85 vs SB5 0.50. Over the entire follow-up, persistence on SB5 was significantly lower vs originator (stratified log-rank p<0.001).	In the PS-matched population, AEs led to discontinuation: SB5 23 (43%) vs originator 3 (6%); loss of response leading to discontinuation: SB5 2 (4%) vs originator 3 (6%). Local skin reactions/pain were common in SB5 (16 patients, 30% of SB5 discontinuations).	Registry with missing FC in many records; relatively small matched sample (n=54 per arm); study population almost exclusively CD.
Jin et al. (2024) [29]	Retrospective study	Retail pharmacy claims representative samples from Germany (84% statutory coverage) and France (45% coverage)	Germany: N=3,362 (54.4% ADA-naïve, 45.6% ADA-experienced). France: N=733 (65.3% naïve, 34.7% experienced).	Initiation of ABP 501 (adalimumab biosimilar); analyzed ADA-naïve vs ADA-experienced subgroups.	Minimum required 365 days pre- and post-ABP501 observation; outcomes reported to 12 months	Median persistence: Germany overall 12.1 mo (95% CI 11.3-13.0); naïve 10.9 mo; experienced 14.2 mo. France overall 12.4 mo (95% CI 10.5-13.9); naïve 12.8 mo; experienced 11.5 mo. 12-month persistence: Germany 50.1% (95% CI 48.4-51.8); France 50.6% (95% CI 46.9-54.2). Switch rates (12 mo): Germany 22.7% switched (most frequently to non-TNFi biologics 41.2% overall; ADA RP 29.0%; ADA biosimilars 16.5%). France 19.8% switched; the majority switched back to ADA RP (81.4% of switches).	No clinical AE or immunogenicity testing reported.	No clinical outcomes reported.
Tursi et al. (2023) [27]	Retrospective, observational, multicenter cohort across study	Adult outpatients with UC or CD who were naïve to or switched to ADA biosimilars.	533 total (162 UC; 371 CD).	ADA biosimilars: ABP501 (Amgevita), SB5 (Imraldi), GP2017 (Hyrimoz), MSB11022 (Idacio). Switches from originator were allowed only if remission ≥1 year.	Median 12 months (IQR 6-24); assessments at 1, 3, 6 mo, then every 6 mo for naïve; switching pts assessed at baseline and every 6 mo	Primary/overall: clinical remission achieved/maintained in 411/533 (77.1%) overall. By biosimilar: ABP501 203/259 (78.3%); SB5 161/214 (75.2%); GP2017 38/49 (77.5%); MSB11022 9/11 (81.8%) (p=0.848). Naïve to biologics:242/304 (79.6%) remission. New to ADA but previously exposed to other biologics: 45/76 (59.2%). Switched (from originator): maintained remission 124/153 (81.0%) - ABP501 83.5%, SB5 78.5%. Clinical response: 479/533 (89.9%). Mucosal healing: 236/344 (68.6%). Steroid-free remission: 456/533 (85.6%). Surgery: 9/533 (1.7%).	AEs: 36/533 (6.7%) had AEs. By agent: ABP501 22/259 (8.5%), SB5 11/214 (5.1%), MSB11022 3/11 (27.3%). Most frequent AE: injection-site itch/pain (predominantly ABP501). Severe AEs: 11/533 (2.1%) (distributed across agents).	Retrospective design, heterogeneous group sizes.

Risk of Bias Assessment

Table 2 shows the risk of bias for prospective and retrospective studies included in the systematic review. Two studies had a low risk of bias [25,28], whereas the other four studies had a moderate risk of bias [19,27,29,30]. In the majority of studies, the risk of bias was minimal in the selection domain. In the comparability domain, a risk of bias was found in most studies, and some studies also had a risk of bias in the outcome domain.

**Table 2:** Risk of bias measured using the Newcastle Ottawa Scale (NOS)

Study	Selection				Comparability		Outcome			Total quality score
	Representativeness of the exposed cohort	Selection of the non-exposed cohort	Ascertainment of exposure	Demonstration that the outcome of interest was not present at the start of the study	Controls for the most important risk factors	Controls for other risk factors	Assessment of outcome	Was the follow-up long enough for outcomes to occur	Adequacy of follow-up of cohorts	
Tapete et al. (2022) [19]	1	1	1	1	0	0	0	1	1	6
Cingolani et al. (2021) [25]	1	1	1	1	1	0	1	0	1	7
Liu Chen Kiow et al. (2024) [30]	1	1	1	0	0	0	1	0	1	5
Lukas et al. (2022) [28]	1	1	1	1	1	1	1	1	0	8
Jin et al. (2024) [29]	1	0	1	1	0	0	1	1	0	5
Tursi et al. (2023) [27]	1	1	1	1	0	0	1	0	1	6

Table 3 shows the risk of bias measured with the JBI tool for the single cross-sectional survey. Jin et al. [26] had an unclear risk of bias in the description of subjects and setting, whereas risk of bias was present in the question related to the measurement of exposure. Confounding factors were also not identified in the study, and no strategies to deal with them were stated.

**Table 3:** Risk of bias measured using the Joanna Briggs Institute (JBI) tool

No.	Question	Jin et al. (2024) [26]
1	Were the criteria for inclusion in the sample clearly defined?	Yes
2	Were the study subjects and the setting described in detail?	Unclear
3	Was the exposure measured in a valid and reliable way?	No
4	Were objective, standard criteria used for measurement of the condition?	Yes
5	Were confounding factors identified?	No
6	Were strategies to deal with confounding factors stated?	No
7	Were the outcomes measured in a valid and reliable way?	Yes
8	Was appropriate statistical analysis used?	Yes

Discussion

Clinical Efficacy and Remission Rates

The findings of this systematic review, based on evidence from seven studies comprising 5721 patients, revealed that switching between reference adalimumab and its biosimilars in IBD does not impact clinical efficacy. Across multiple real-world studies, patients initiating or switching to biosimilar adalimumab (SB5 or ABP501) achieved high rates of clinical remission or response comparable to those maintained on the originator product. For example, Cingolani et al. found that among IBD patients who switched to ABP501, 85.5% were in remission at baseline and 76.4% remained in remission at six months (p=0.09) [25]. In the smaller SB5 switch cohort (n=25), remission declined from 96% to 84% (p=0.20) over six months [25]. Lukas et al. reported no significant difference in Crohn’s disease activity between two propensity-matched cohorts - one switched to SB5 and the other continued the originator - through 52 weeks [28].

In a large multicenter cohort by Tursi et al., about 80% of biosimilar-treated patients, both biologic-naïve and switchers, were in clinical remission, and similar remission rates were observed for ABP501 and SB5 (75-78%) [27].

These findings are further supported by Regueiro et al., who compared five adalimumab biosimilars (ABP501, SB5, MSB11022, GP2017, and FKB327) in bio-naïve IBD patients across multiple centers, confirming comparable effectiveness regardless of which biosimilar was used [31]. Collectively, these findings align with previous literature: Kay et al. reported that switching does not reduce efficacy at the group level, and real-world evidence consistently shows SB5 to be as effective as its reference product [32].

Biomarker Outcomes

In another review that assessed the efficacy of switching between reference adalimumab and biosimilars in inflammatory diseases, including IBD, it was revealed that efficacy in switching groups was comparable to continuous biosimilar and continuous reference arms, with no significant differences in treatment-emergent AEs, anti-drug antibodies, or neutralizing antibodies [17]. Similarly, in the present systematic review, biomarker outcomes generally paralleled clinical results. Most studies reported stable or modestly improved inflammatory markers after switching. For instance, Tapete et al. found that CRP and FC levels remained essentially unchanged in patients switched to SB5 [19].

Similarly, Cingolani et al. observed no significant change in median FC or CRP-positivity after switching to ABP501, whereas in the SB5 arm, median FC fell (97 μg/g → 50 μg/g) and the proportion with positive CRP decreased significantly (48% → 20.8%; p=0.03) [25].

Lukas et al. noted no meaningful difference in CRP or FC between SB5 switchers and originator-maintainers through 52 weeks [28]. Overall, evidence showed that objective inflammation markers generally did not worsen after switching and often remained stable. Wiland et al., in their study, also reported similar findings as observed in the current systematic review. They reported no significant difference regarding CRP after switching to biosimilar SDZ-ADL [33].

Treatment Persistence and Drug Survival

Treatment persistence and drug survival varied by study setting. Long-term persistence tended to be high and often comparable between switchers and non-switchers. For example, Liu Chen Kiow et al. observed no significant difference in 30-month drug persistence between patients switched to biosimilars and those remaining on Humira (83.7% vs. 88.2%, log-rank p=0.543) [30]. Cingolani et al. reported similar six-month survival (p=0.20) between ABP501 switchers and originator-maintainers [25]. These findings suggest that when care is tightly managed in specialized centers, persistence on therapy remains high after switching. However, broader real-world data paint a more heterogeneous picture. Pharmacy claims analyses in large populations found that roughly half of ABP501-treated patients remain on therapy at one year [29]. However, patients with prior adalimumab exposure tended to switch back to the originator or another biologic more often than naïve patients [29]. Lukas et al. reported that one-year persistence was 87% on originator versus only 65% on SB5 (p<0.001) [28]. Most SB5 discontinuations in that study were due to injection-site reactions. Thus, persistence tends to be higher in controlled clinical cohorts and lower in unselected populations, reflecting differences in patient selection, follow-up intensity, and healthcare policies. Similar to the present systematic review, a prospective study that assessed acceptance and persistence of adalimumab biosimilars in IBD patients found a 91.8% acceptance rate [34]. Persistence with the initial biosimilar was 68.6% at six months and 60.4% at 12 months, while the survival without biosimilar discontinuation was 76.7% at six months and 71.0% at 12 months [34]. As in the current systematic review, injection-site pain (24.7%) was the leading cause of discontinuation, and 22 patients switched back to the originator, yet clinical remission was maintained in 90.4% at 12 months [34]. Consistent with this, Fernández-Cano et al. reported real-world persistence data for adalimumab originator and biosimilar in IBD, further corroborating the broadly comparable drug survival observed across switching and continuation groups [35].

Safety and Tolerability

Safety and tolerability were consistently reassuring. Serious AEs were uncommon across all studies. Tursi et al. found only 2.1% of patients experienced severe AEs over 12 months [27], and Cingolani et al. reported only one discontinuation for AEs per biosimilar cohort over six months [25]. The most frequently reported side effects were mild local injection-site reactions. For instance, Tapete et al. reported injection-site pain in 24.7% of patients, predominantly among switchers [19]. In the Lukas et al.'s SB5-switch cohort, 30% of SB5 discontinuations were due to local skin reactions or pain [28]. Tursi et al. similarly found that injection-site itching or pain, mostly ABP501, accounted for the majority of non-serious AEs [27]. Aside from these local reactions, no consistent safety signals emerged. Immunogenicity data are sparse in the switching literature. However, Kay et al., in their review, reported that clinical trials showed comparable immunogenicity [32]. Importantly, the multicenter SUSTAIN (Safety and Effectiveness of Multi-Switch Between Adalimumab Originator and Biosimilars) study by Shehab et al. examined safety and effectiveness specifically in multi-switch scenarios between adalimumab originator and biosimilars, finding no new safety signals even after sequential switching - a clinically relevant finding as multi-switch practices become more common in formulary-driven healthcare environments [36].

Strengths and Limitations

This systematic review is the first to synthesize and critically evaluate available evidence specifically on switching between reference adalimumab and its biosimilars in adult patients with IBD. A comprehensive and sensitive literature search across four major databases ensured broad coverage of published evidence. The review included more than 5700 patients across diverse study designs, populations, and biosimilars, strengthening the generalizability of the findings. Both clinical outcomes and objective biomarkers were considered, alongside persistence, safety, and immunogenicity.

However, there are certain limitations as well that should be acknowledged. The available evidence was heterogeneous in terms of study design, patient populations, biosimilars assessed, and outcome definitions, limiting direct comparability. Most studies were observational, with only a minority being prospective in design, and several had a moderate risk of bias, particularly in the comparability and outcome domains. Follow-up durations varied widely, with some studies reporting only short-term outcomes (≤6 months), which restricts conclusions on long-term safety, persistence, and immunogenicity. Baseline disease severity, disease location, and behavior (e.g., stricturing or penetrating phenotype in Crohn’s disease) were inconsistently reported across included studies, limiting assessment of whether outcomes varied by these clinically important subgroups. Furthermore, outcome definitions were heterogeneous: while most studies relied on clinician-reported remission scores and biomarkers (CRP, FC), mucosal healing was reported in only a subset of studies, and histological remission was rarely assessed - an important gap given the growing emphasis on deep remission as a treatment target. Safety reporting was inconsistent and immunogenicity testing was sparse, preventing firm conclusions about rare AEs or antibody development. Publication bias could not be excluded, and real-world studies often lacked standardized outcome reporting, particularly for mucosal healing, steroid-free remission, and surgery.

Implications for Practice and Research

The findings support that switching from reference adalimumab to biosimilars does not compromise clinical efficacy or safety for most patients with IBD. Reported remission and persistence rates in switchers were broadly comparable to those maintained on originator therapy, which suggests that non-medical switching can be considered a viable strategy in clinical practice. Injection-site reactions remain the most frequent tolerability issue and should be closely monitored, as they may influence persistence in some patients. Clinicians should provide clear communication with patients to maintain confidence in biosimilar use and ensure adherence.

Future studies should prioritize prospective, adequately powered designs with longer follow-up to evaluate persistence, immunogenicity, and long-term safety outcomes. Standardized definitions of remission, response, and persistence would allow for better cross-study comparison. Real-world studies should systematically collect data on objective outcomes such as mucosal healing, hospitalizations, and surgery, alongside patient-reported outcomes and quality of life.

## Conclusions

This systematic review provides a comprehensive synthesis of the available evidence on switching between reference adalimumab and its biosimilars in adult patients with IBD. Across diverse study designs and healthcare settings, clinical effectiveness following a switch appears broadly comparable to continued use of the originator, with remission and response rates maintained in the majority of patients. Biomarker stability and favorable outcomes such as mucosal healing and steroid-free remission reinforce this finding, although data remain limited in scope and uniformity. Safety outcomes were consistent across studies, with no new or unexpected AEs identified. Local injection-site reactions were the most frequent tolerability issue and, in some cohorts, the leading reason for discontinuation. Serious AEs were uncommon. Future research should prioritize standardized immunogenicity assessments, long-term outcomes, and comparative cost-effectiveness to strengthen confidence in interchangeability.
